# Implementing Advance Choice Documents as Part of Routine Mental Health Care

**DOI:** 10.1192/bjo.2026.11170

**Published:** 2026-06-30

**Authors:** Mariam Namasaba, Rajesh Mohan, Mandy Mudholkar, Shubulade Smith, Claire Henderson

**Affiliations:** 1 King’s College London, London, United Kingdom; 2 South London and Maudsley NHS Foundation Trust, London, United Kingdom

## Abstract

**Aims:**

Increasing rates of involuntary treatment in the UK and persisting ethnic disparities in detentions have led to the reform of the Mental Health Act (MHA) in 2025. The MHA bill introduces several new measures including the offer of Advance Choice Documents (ACDs) to people who wish to make them. ACDs are written statements of a person’s wishes and preferences for treatment and care, made when a person is well and has the capacity to do so. Systematic reviews show that ACDs can reduce detention rates by up to 25%. The South London and Maudsley NHS Foundation Trust (SLaM) rolled out ACDs as part of routine care, ahead of the MHA 2025. This study describes the implementation of ACDs as part of routine care at SLaM.

**Methods:**

SLaM recruited independent senior mental health professionals (ACD facilitators) to lead the creation, use and review of ACDs. The facilitators underwent training and utilised resources previously developed through previous research. The resources included an ACD template, an ACD manual and role description for facilitators, simulation training and Recovery College courses. A clinical lead and clinical supervisor were also employed to provide oversight for the implementation project. Data on implementation were collected through case note reviews, and interviews with service users and staff. Analyses on how ACDs were created and used when people experienced crises were conducted.

**Results:**

At the time of abstract submission, 77 ACDs have been created with service users at SLaM. The implementation of ACDs has been aided by service user-led initiatives such as the Patient Carer Race Equality Framework, staff training, and Recovery College courses which enabled people to create ACDs with high fidelity to the evidenced interventions. Staff and service users report that the ACD creation process is a holistic experience that is empowering and beneficial for improving therapeutic alliance between service users and mental health professionals. The main barrier cited by staff was a lack of time due to large caseloads.Service users expressed scepticism about their ACDs being accessed and honoured during times of crisis.

**Conclusion:**

Implementing evidence-based interventions in healthcare settings is complex. SLaM has replicated the implementation of ACDs with high fidelity to the intervention trials that reported reductions in Mental Health Act detentions and greater autonomy for service users. Our results show that multiple strategies are needed to accelerate the reach and adoption of ACDs in other Trusts.

